# Molecular Initiating Events Associated with Drug-Induced Liver Malignant Tumors: An Integrated Study of the FDA Adverse Event Reporting System and Toxicity Predictions

**DOI:** 10.3390/biom11070944

**Published:** 2021-06-25

**Authors:** Kota Kurosaki, Yoshihiro Uesawa

**Affiliations:** Department of Medical Molecular Informatics, Meiji Pharmaceutical University, 2-522-1 Noshio, Kiyose, Tokyo 204-8588, Japan; d196955@std.my-pharm.ac.jp

**Keywords:** FAERS, molecular initiating events, adverse outcome pathways, drug-induced liver injury, liver cancer, machine learning

## Abstract

Liver malignant tumors (LMTs) represent a serious adverse drug event associated with drug-induced liver injury. Increases in endocrine-disrupting chemicals (EDCs) have attracted attention in recent years, due to their liver function-inhibiting abilities. Exposure to EDCs can induce nonalcoholic fatty liver disease and nonalcoholic steatohepatitis, which are major etiologies of LMTs, through interaction with nuclear receptors (NR) and stress response pathways (SRs). Therefore, exposure to potential EDC drugs could be associated with drug-induced LMTs. However, the drug classes associated with LMTs and the molecular initiating events (MIEs) that are specific to these drugs are not well understood. In this study, using the Food and Drug Administration Adverse Event Reporting System, we detected LMT-inducing drug signals based on adjusted odds ratios. Furthermore, based on the hypothesis that drug-induced LMTs are triggered by NR and SR modulation of potential EDCs, we used the quantitative structure–activity relationship platform for toxicity prediction to identify potential MIEs that are specific to LMT-inducing drug classes. Events related to cell proliferation and apoptosis, DNA damage, and lipid accumulation were identified as potential MIEs, and their relevance to LMTs was supported by the literature. The findings of this study may contribute to drug development and research, as well as regulatory decision making.

## 1. Introduction

Drug-induced liver injury (DILI) is the most common adverse drug event (ADE) causing medicinal product withdrawal or major regulatory action [1]. More than 1100 products used by humans on a relatively frequent basis, such as medicines, herbal and other natural products, minerals, and “recreational” or illicit chemical substances, have been identified as potential liver injury-inducing substances [2].

As described by Katarey et al., liver malignant tumors (LMTs) represent a serious adverse event associated with DILI [3]. DILI-induced hepatocellular death leads to clinical manifestations of hepatitis and can induce hepatic malignancies if DILI progresses. Due to the regulatory implications and potential for substantial impacts of drug-induced LMT on patients’ lives, early detection of LMT-inducing drugs and developing an understanding of the compound’s biological interactions are important considerations across all phases of pharmaceutical development.

LMTs associated with the use of steroids, e.g., anabolic-androgenic steroids (AAS) and oral contraceptives (OC), have been sporadically reported [4,5,6,7,8]. AAS and OC are a typical class of potential endocrine-disrupting chemicals (EDCs); therefore, the mechanism of drug-induced LMTs can rely on the modulation of function through nuclear receptors (NRs) and stress response pathways (SRs), which are triggered by drugs belonging to a class of potential EDCs.

EDCs interfere with the endocrine system by interacting with NRs and stress response pathways (SRs), engendering a myriad of adverse developmental, reproductive, neurological, and immunological effects in both humans and wildlife [9,10]. Among chemical exposures that negatively affect the liver, the growing EDC class has gained attention due to its hepatic function-disrupting potential [11]. Exposure to EDCs leads to lipid accumulation in the liver, which is the first (reversible) stage of nonalcoholic fatty liver disease (NAFLD). In the liver, the activation and/or recruitment of macrophages results in nonalcoholic steatohepatitis (NASH) and eventual fibrosis. A subset of cases culminates in the development of neoplastic events that give rise to hepatocellular carcinoma (HCC), which is the final endpoint of hepatic disease progression [12]. Therefore, we cannot ignore the potential for serious liver malignancy adverse events, caused by drugs identified as potential EDCs; developing an understanding of the drug classes at risk for LMT induction and mechanisms of compound–biological interactions leading up to LMT in the toxicological framework is essential.

Adverse outcome pathways (AOPs) [13] provide a framework to understand the linkages between toxicological insights and meaningful endpoints for ecological risk. AOPs represent the sequence of events, from chemical receptor activation to in vivo adverse outcomes (AO), via a series of key events at cellular and subcellular levels.

Molecular initiating events (MIEs)—often crucial steps in AOPs—are generally positioned as the initial interaction between a molecule and a biomolecule or biosystem that can be causally linked to an outcome via an AOP [14]. MIEs are a concept that arose from the demand to understand the chemical and biological mechanisms behind the toxicological endpoints triggered by substances. MIE chemistry elucidation is essential for developing predictive methods, such as structure–activity relationships and quantitative structure–activity relationships (QSAR), and can also facilitate AO predictions based on MIE activities, as demonstrated by Gadaleta et al. [15] for steatosis. Therefore, understanding MIEs is a practical issue in modern toxicology.

However, our understanding of the drug classes at risk for LMT induction, and MIEs that are specific to drugs that develop these adverse events, is limited due to at least three of the following factors: 1) rare cases of drug-induced LMT are difficult to evaluate in the limited population of clinical trials, impeding attempts to identify drug classes associated with LMT; 2) the pathogenesis of drug-induced hepatic malignancies can be multi-step, multi-disease processes with diverse chemical etiologies, preventing the identification of MIEs associated with LMT; and 3) comprehensively evaluating the MIEs of drug groups associated with LMT, via in vitro or in vivo screening, is unrealistic in terms of time and cost efficiency. Therefore, prioritizing the MIEs associated with drug-induced LMT proves difficult, limiting the availability of useful data for drug development, research, and regulatory decision making.

Alternatively, in silico technologies, such as computational toxicology, provide considerably cheaper and faster approaches that also circumvent the ethical and cost issues related to animal models. Moreover, such approaches cohere with the current tendency to replace non-clinical tests with in vitro or in silico alternatives, mandated by the implementation of the “3R” principle [16]. Additionally, the United States Food and Drug Administration (FDA) developed the FDA Adverse Events Reporting System (FAERS), which is a spontaneously reported ADE database that contains over 10 million publicly available ADE cases. FAERS data can be applied for the surveillance of post-market drug safety; several researchers have successfully identified drug safety signals through FAERS analyses [17,18]. The application of data-driven FAERS or computational toxicology methodologies could provide a breakthrough for overcoming the challenges associated with the evaluation of LMT-inducing drugs and their toxicological properties.

The purpose of this study was to examine potential MIEs that are specific to LMT-inducing drugs, using an ADE database and QSAR prediction for chemical toxicity. We detected signals of drugs associated with LMT using FAERS. In this study, LMT-inducing drugs were evaluated based on adjusted odds ratios (aOR), accounting for the possibility that the therapeutic purposes (i.e., patient background diagnoses) of the drugs may be confounding factors in the signal. Moreover, we hypothesized that drug-induced LMTs were triggered by the NR and SR activities of drugs belonging to the class of potential EDCs. We calculated the agonist/antagonist activities of signal-detected drugs against 56 NRs and SRs, using Toxicity Predictor [19], which is a QSAR prediction platform for chemical toxicity, and, using these activities, we identified potential MIEs that are specific to the class of signal-detected LMT-inducing drugs.

## 2. Materials and Methods

### 2.1. Study Overview

**Figure 1:**
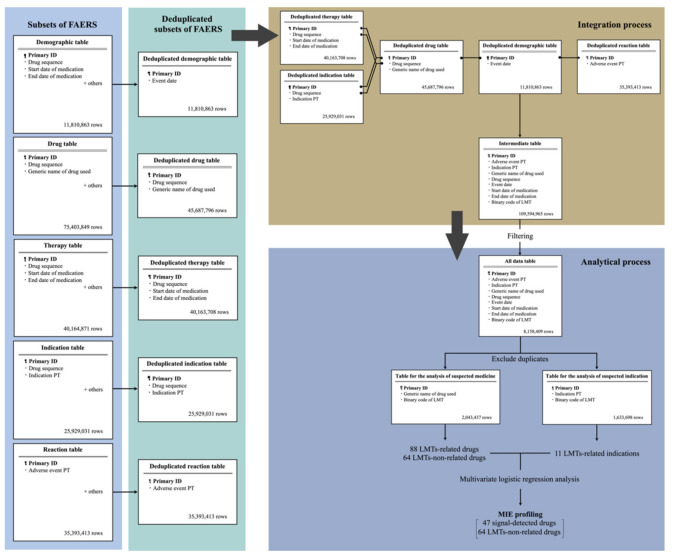
Overview of this study. Five subsets of the FAERS database were deduplicated (the square of deduplicated subsets of FAERS). Deduplicated five subsets were merged and integrated into one table (integration process), and information such as start date of medication, end date of medication, and event date were filtered. All information that could be a reason for pseudo-signal were excluded. “All data table” (>8 million) was used for the detection of LMT-inducing drugs and LMT-related indications, and signal-detected drugs were evaluated in detail with multivariate analysis. Finally, MIEs specific to signal-detected drugs were calculated (analytical process).

We used the following five subsets of the FAERS database: demographic table, therapy table, indication table, drug table, and reaction table. These subsets were deduplicated, merged, and integrated into one table (intermediate table) (Figure 1). Our study design was complete case analyses. To extract complete and consistent patient information from FAERS, start date of medication, end date of medication, and event date were filtered and information that can be a cause of pseudo-signal were excluded. The filtered “All data table” (>8 million) was used for the detection of LMT-inducing drugs and LMT-related indications. Based on the results, multivariate logistic regression was performed, and aORs of LMT-inducing drugs were determined. Finally, MIE profiling was performed to determine agonist/antagonist activities of MIEs for signal-detected drugs and non-LMT-inducing drugs.

### 2.2. Database

The FAERS database is medical big data, created to support FDA’s post-marketing surveillance on drugs and therapeutic agents [20]. FAERS contains adverse event and medication error reports submitted to the FDA via MedWatch, FDA Safety Information, and the Adverse Event Reporting Program. ADEs and indications (diagnoses) reported in FAERS were described using preferred terms (PTs) defined in the Medical Dictionary for Regulatory Activities (MedDRA) [21]. MedDRA is a systematized medical thesaurus that facilitates the search and regulation of medical terms in medical data.

To simplify access to chemical structural formulas corresponding to the generic names of drugs, we used a version of FAERS that facilitates the retrieval and management of generic names and CAS registry numbers^®^. The corresponding medical product names and generic names, or the corresponding generic names and CAS registry numbers^®^ were obtained from the WHODrug [22] master data created by Uppsala Monitoring Center. The curation of medical product names, generic names and CAS registry numbers^®^ were conducted by INTAGE Healthcare Inc. (Tokyo, Japan, https://www.intage-healthcare.co.jp/, accessed on 25 June 2021). The FAERS database included 35,393,413 reports of ADEs recorded from 1 January 2004 to 31 March 2020. The FAERS database is composed of seven subsets of data tables, namely, demographic, therapy, indication, drug, reaction, outcome, and report source tables. The drug table was linked to the therapy and indication tables along with both the primary ID and drug sequence; the demographic table was linked to the drug, reaction, outcome, and report source tables along with the primary ID [23].

### 2.3. Definition of PTs Associated with LMTs

For signal detection of LMT-related ADEs, we grouped PTs in the FAERS database to define the set of PTs subject to a disease group associated with LMT. The PT set and LMT correspondence were defined according to the narrow scope of Standardized MedDRA Queries version 22.0 (SMQ ver. 22.0) [24]. However, we detected additional PTs in the FAERS database that were related to LMT, but not recorded in the SMQ. Therefore, we added the following five PTs to LMT-related PTs defined with SMQ: carcinoma hepatocellular, hepatic neoplasm malignant, hepatic neoplasm malignant non-resectable, hepatic neoplasm malignant resectable, and hepatic neoplasm malignant recurrent. Table 1 shows the set of 21 PTs defined as the disease group associated with LMTs.

### 2.4. Patient Information Extraction

We created a data table that integrated information on patients, drugs used, purpose of the drugs used, medication start date, reported ADEs, AED report date, and medication stop date. We also created a suspected drug analysis table and a suspected applicable disease (diagnosis) analysis table (Supplemental Information). First, duplicate records were eliminated for each of the following five subsets: demographic table, therapy table, indication table, drug table, and reaction table. Next, these deduplicated five subsets were merged and integrated into a table. To extract reports from the integrated table that were consistent with the reported ADEs and the drug treatment time series, we extracted only records that satisfied the condition that the ADE report date was within the period between the medication start and end dates. In addition, in some cases, the indications of the drugs used were reported as ADEs owing to insufficient efficacy of the drug used for that therapeutic purpose, which could cause a pseudo-signal [25] for drug-induced LMTs. Therefore, when records had PTs of indications that belonged to the set of LMT-related PTs, the records were excluded from the data table. Finally, binary information was added to the table to indicate whether the ADE PT was an LMT-related PT. After these adjustments, the “All data table” was generated (8,158,409 rows). Furthermore, to examine drugs suspected of causing LMTs or indications (diagnoses), two data tables, “Table for the analysis of suspected medication” and “Table for the analysis of suspected indication,” were created from the “All data table”. An example of a preprocessed FAERS database is available in Supplemental Material 1.

### 2.5. Reporting Odds Ratio Calculations

The reporting odds ratio (ROR), used in pharmacovigilance to detect ADE signals, can be calculated based on two-by-two contingency tables [26]. We analyzed LMT-related drug signals for all drugs listed in the preprocessed FAERS data table described in Section 2.3. In this signal analysis, the LMT-related drug reporting rate was assessed by ROR based on the following equations (Equations (1) and (2)) and contingency table (Table 2). Similarly, for the signal detection of LMT-related indications (diagnosis), RORs for all indications listed in the preprocessed FARES data table were assessed. The two-by-two contingency table cannot be calculated with 0 cells, and the estimation is unstable when the cell frequency is small. Hence, 0.5 was added to all cells as a correction (Haldane–Anscombe half correction) [27,28]. We used Fisher’s exact test to assess the independence of the associations of drugs/indications and LMT-related ADEs. The significance level was set at 0.05. The threshold ROR and *p-*values to identify signal-detected LMT-related drugs and LMT-related indications were >1 and <0.05 (by Fisher’s exact test), respectively.

(1) RORdrug=adrugbdrugcdrugddrug

(2) RORdrug=aindbindcinddind

The signal-detected LMT-related drugs and LMT-related indication diseases (diagnoses) included reports of drugs with statistical significance and high ROR, but very few cases of drug use, as well as reports of indication diseases with statistical significance and high ROR, but very few cases of treatment.

Therefore, lower limits were applied to both the number of drug use reports and the number of indication reports to detect more reliable signals. This approach was adopted because there were at least two disadvantages for further filtering based on only *p*-values as reliability indicators. First, if the significance level with a *p-*value of <0.05 is met, further *p*-value comparisons will not provide statistically useful information. In that case, the effect size (ROR) rather than the *p*-value should be utilized. Second, if a threshold based on *p*-values is introduced, information on frequently used drugs and frequently conducted treatments might be overlooked. In that case, further comparisons of *p*-values large and small for factors with statistical significance would not provide useful information. Furthermore, signals of frequently used drugs could be overlooked although they meet the requirement of a *p*-value of <0.05. Therefore, we introduced a threshold for the number of reports to ensure that frequently used drugs and indications were not overlooked, and to screen relevant drugs and indications.

The threshold for the lower limit was defined according to the value of a + b in the contingency table, i.e., the total number of reports on the use of a specific drug or treatment performed under a specific indication. A threshold value of 99 was adopted for LMT-related drug signal detection, and a threshold value of 200 was adopted for LMT-related indication signal detection. These thresholds were mechanically determined to ensure that 99% of the drugs and indications with a *p*-value of <0.05 and an ROR of >1 were included in the cumulative number of reports of all drugs or indications.

### 2.6. Graphical Summaries (Volcano Plot)

Volcano plots are effective and easy-to-interpret graphs that summarize both fold-changes and statistical significance to visualize trends in large datasets [29]. Some studies have applied volcano plots to adverse event databases [30,31,32,33]. To identify trends in exposure factors associated with ADEs, volcano plots were applied to the FAERS data. We plotted the natural logarithms of RORs (lnROR) on the abscissa axis and common logarithms of the inverse *p-*value obtained via Fisher’s exact test on the vertical axis, respectively. Utilizing volcano plots, the trends of each drug and indication associated with LMT induction in the FAERS database were visualized exhaustively.

### 2.7. Selection of Indications for Multivariate Logistic Regression Model Covariates

From 32 indications (Table S1) with suspected associations with LMTs identified in the volcano plot, we selected 11 indications to incorporate as covariates in the multivariate logistic regression model. The indications were selected from patient background information based on whether the indication (diagnosis) was generally accepted as a risk factor for LMT based on literature reviews (Supplemental Information) [34,35,36].

### 2.8. Multivariate Analysis

The results presented in Section 3.2 were used for the multivariate analysis. To evaluate adjusted ROR (aOR), accounting for the effect of drugs and indications (diagnoses) on the signals of LMT-related drugs, we built a multivariate model containing the drug and indication classes discussed in Section 3.2. From indications detected in the univariate analysis, 11 indications that were determined to have relationships with LMT and 88 signal-detected drugs were adopted for multivariate analysis. The use of all 88 drugs detected in the univariate analysis and the presence of a treatment subject to each of the 11 indications were encoded as binary values of 0 or 1 for each primary ID, respectively. To detect multicollinearity among 99 variables adopted for the multivariate analysis, Pearson’s correlation coefficients were calculated for all pairs of encoded variables. Multicollinearity was defined as pairs of variables with *R*^2^ values of 0.8 or higher. For such pairs, the variable with a higher correlation with the objective variable (binary value of [0,1] encoded against LMT reporting) was adopted for the multivariate analysis. The drug and indication variables redefined in the multicollinearity detection were carried forward for multivariate logistic regression modeling. Finally, in the constructed multivariate logistic regression model, items of variables that were not significant, subject to the contribution for the association to the outcome, were excluded based on the *p*-value of the Wald test, and a final multivariate logistic regression model was reconstructed using only significant variables. The significance level of partial regression coefficient was set at 0.05 based on the Wald test.

### 2.9. Dataset for MIE Activity Predictions

To evaluate the toxicity response profiles of drugs identified as significant predictors of LMT in the multivariate logistic regression model (described in Section 3.3 and Section 2.6), a new group of compounds was established. The compound population (n = 111) for analysis comprised 47 drugs with the following processing added to the 48 drugs that served as significant predictors in the multiple logistic regression model and 64 drugs that did not induce LMT. The following processing was applied to the list of 48 drugs: (1) the 48 drugs that were identified as significant predictors of LMT in the multivariate logistic regression model had the excluded pairs that exhibited multicollinearity; these drug pairs were added to the compound group again, and (2) high-molecular-weight compounds such as antibody drugs were removed from the group of compounds. Conversely, the non-LMT-inducing drugs comprised 64 drugs detected under the condition of an lnROR <0 and a *p* of <0.05, based on the volcano plot used to detect the signal of LMT-inducing drugs. Finally, 111 compounds were analyzed for toxicity response profiles. A complete list of Simplified Molecular Input Line Entry System (SMILES) structures and predicted MIE activities for all 111 compounds is available in Supplemental Material 2.

### 2.10. MIE Activity Prediction Using QSAR Toxicity Predictor

For each compound in the group established for evaluating MIE activities, chemical structure information was provided in the SMILES format. To predict MIE activities, we used Toxicity Predictor [19], our previously developed QSAR platform based on machine learning models trained on the Tox21 10K compounds library to evaluate agonist and antagonist activity against MIEs. Through the Toxicity Predictor, the SMILES strings were cleaned and standardized (removing salts, counterions and fragments, and adjusting the protonation state (neutralized)), optimal 3D conformers were determined, and MIE activity predictions were conducted based on molecular descriptors calculated from the optimal conformer. We calculated the agonist and antagonist activity values (MIE activity) of 56 NR and SR pathways for each of the 111 compounds. Cutoff values for predicted MIE activity were calculated using the Youden method [37], and the calculated MIE activities were normalized to obtain a cutoff value of 0.5. Therefore, the predicted labels of compounds with values above 0.5 as normalized predicted values were converted to 1, and those with predicted values below 0.5 were converted to 0.

### 2.11. Statistical Profiling of LMT-Inducing Drug MIE Activities

The compound group established as LMT-inducing or non-LMT-inducing drugs in Section 2.7 was coded as a binary value of [0,1] to examine whether there was a significant difference in MIE activity values between LMT-inducing and non-LMT-inducing drugs. Using the prediction labels for each MIE activity calculated using Toxicity Predictor (i.e., values encoded in binary labels of [0,1] and binary labels encoding the class of LMT-inducing drugs summarized in a cross-tabulation table), we determined the ROR of LMT-inducing drugs against non-LMT-inducing drugs, using drug MIE activities as an exposure factor (Equation (3); Table 3). We used Fisher’s exact test to assess the independence of associations of MIE activities and LMT-inducing drugs; the significance level was set at 0.05. The criteria for signals used in this analysis included an ROR of >1 and a *p*-value of <0.05 (calculated with Fisher’s exact test).

(3) RORMIE=aMIEbMIEcMIEdMIE

### 2.12. Comparison with the Latest DILIst Dataset

The DILIst dataset, which is an expansion of the DILIrank dataset, is annotated using the US FDA drug labeling [38], and the latest database contains 1279 DILI-positive and DILI-negative drugs [39]. To examine the association of signal-detected LMT-inducing drugs with known DILI, we compared our MIE dataset (n = 111, described in Section 2.9) with the latest DILIst dataset. First, for all drugs in the MIE dataset, the percentage of overlap with all drugs in the DILIst dataset was calculated. In addition, the overlap rates of DILI-positive drugs in the DILIst dataset were calculated for the class of LMT-inducing drugs in the MIE dataset. Next, we analyzed the drug categories of all drugs in the MIE and DILIst datasets. The Anatomical, Therapeutic, Chemical (ATC) Classification System, a system of classification of medicines maintained by the World Health Organization and accepted internationally, was used for the drug classification [40]. In the ATC Classification System, drugs are divided into five levels [41]. The highest level is the main anatomical group, the second level is the therapeutic subgroup, the third level is the pharmacological subgroup, the fourth specifies chemical subgroup, and the fifth level denotes chemical substance [42,43]. We linked the anatomical and therapeutic subgroups of the ATC classification to both the MIE and DILIst datasets and analyzed their trends of occurrence.

### 2.13. Software

All data were analyzed using JMP Pro 16.0 (SAS, Cary, NC, USA).

## 3. Results

### 3.1. Presentation of Specimen Population Data for Analysis

The demographic table included 11,810,863 rows, the drug table included 75,403,849 rows, the reaction table included 35,393,413 rows, the therapy table included 40,164,871 rows, and the indication table included 25,929,031 rows. These tables were integrated into a single preprocessed FAERS data table that included 8,158,409 rows; 2496 LMT records and 1232 unique LMT records; 1,405,540 unique ADE reports; 4233 unique drug reports; and 10,240 unique indication reports.

**Figure 2:**
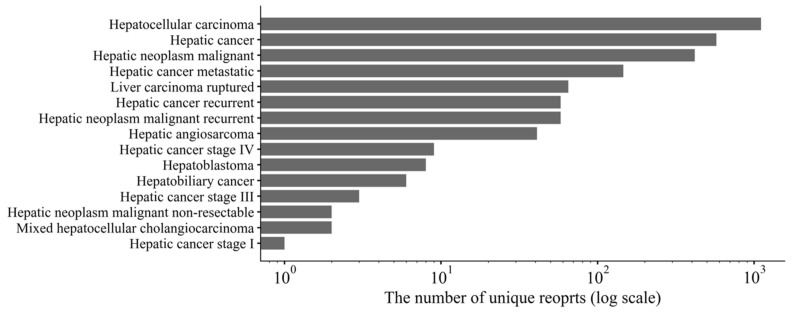
Numbers of unique records of MedDRA preferred terms (PTs) related to liver malignant tumors.

The numbers of unique records with PTs related to LMT ADEs are shown in Figure 2. The three most common PTs in the records were hepatocellular carcinoma (44%), hepatic cancer (23%), and hepatic neoplasm malignant (17%).

### 3.2. LMT-Related Drug and Indication Signal Detection

**Figure 3:**
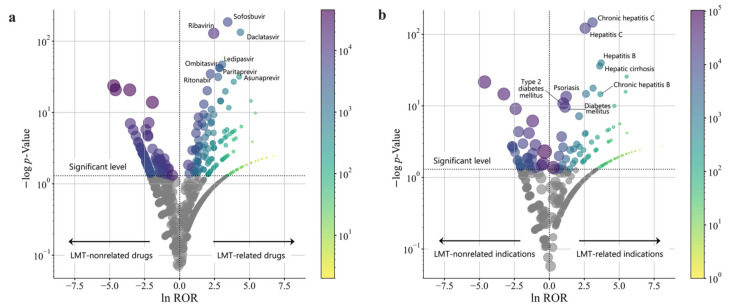
Drugs (**a**) and indications (**b**) associated with the development of LMT. The abscissa axis shows the natural logarithm of reporting odds ratios (lnROR); the vertical axis shows the common logarithm of inverse *p*-values (−log10(*p*)) obtained by Fisher’s exact test. The dotted line on the y-axis represents a *p*-value of 0.05. The marker size and color spectrum from yellow to purple correspond to a + b from the contingency table, i.e., the number of reports of the use of the drug or the indication (diagnosis).

For each drug or indication in the preprocessed FAERS database, these two statistics were plotted exhaustively on each volcano plot (Figure 3). Drugs or indications with highly significant differences and higher RORs, as indicated by the higher values of the logarithmically transformed inverse *p*-values, were represented with higher positive values on the vertical axis and depicted in the upper right portion of the volcano plot. Based on the definitions of signals of LMT-related drugs and the indications described in Section 2.5, 133 drugs and 78 indication diseases were signal-detected.

Using the threshold described in Section 2.5, the number of signal-detected LMT-related drugs was reduced to 88 (summarized in Table S2). Among 88 drugs, the 10 drugs with the highest RORs were “Daclatasvir,”, “Asunaprevir,”, “Entecavir”, “Cinnamomum cassia”, “Adefovir”, “Sofosbuvir”, “Quinine”, “Panax ginseng”, “Atractylodes lancea”, and “Velpatasvir” (Table 4). The major drug classes to which these drugs belong are anti-HCV drugs, anti-HBV drugs, and Chinese herbal medicines.

Using the threshold, the number of signal-detected LMT-related indications (diagnoses) was reduced to 32 (summarized in Table S1). Among 32 indications, the 10 with the RORs were “hepatitis B”, “chronic hepatitis B”, “hepatic cirrhosis”, “liver transplant”, “chronic hepatitis C”, “prophylaxis against transplant rejection”, “immunosuppressant drug therapy”, “hepatitis C”, “fluid retention”, and “liver disorder” (Table 5). The major indication classes to which these indications belong are infectious liver disease, caused by HCV and HBV, hepatic cirrhosis, and other liver diseases.

As shown in Figure 3a and Table 4, most drugs were for the treatment of infectious liver diseases, suggesting that underlying liver diseases were potential confounding factors.

The LMT-related indication results were further examined via a literature review (described in Section 2.7), to determine whether the patient background of being treated under the indication was related to the risk of LMT development. The following eleven indications that were strongly related to the risk of LMT development were identified: “hepatitis B”, “chronic hepatitis B”, “hepatic cirrhosis”, “chronic hepatitis C”, “hepatitis C”, “liver disorder”, “growth hormone deficiency”, “psoriasis”, “diabetes mellitus”, “type 2 diabetes mellitus”, and “hyperlipidemia”. These indications belonged to the main classes of infectious liver disease and hepatic cirrhosis. Other classes of indications that were strongly related to LMT development were metabolic syndromes, such as hyperlipidemia, diabetes, and endocrine diseases, which were possible risk factors for liver cancer associated with NAFLD [44].

### 3.3. Multivariate Analysis for Drugs and Indications Suspected of Causing LMTs

As shown in Section 3.2, the major class of drugs suspected to induce LMTs consisted of the drugs used for the treatment of infectious liver diseases. Additionally, signal detection of indications associated with LMT induction identified patient backgrounds related to infectious liver diseases, hepatic cirrhosis, diabetes, metabolic syndrome, and endocrine diseases, which are risk factors for the development of hepatocarcinoma. These results suggest that therapeutic drugs prescribed to patients with backgrounds of a high risk of developing LMTs are signal-detected as LMT-inducing drugs. Therefore, we conducted a multivariable logistic regression to obtain aORs for 88 drugs and the 11 indications detected in Section 3.2, considering the possibility that patient backgrounds and the drugs used represent potential confounders in LMT-inducing drug screening.

**Figure 4:**
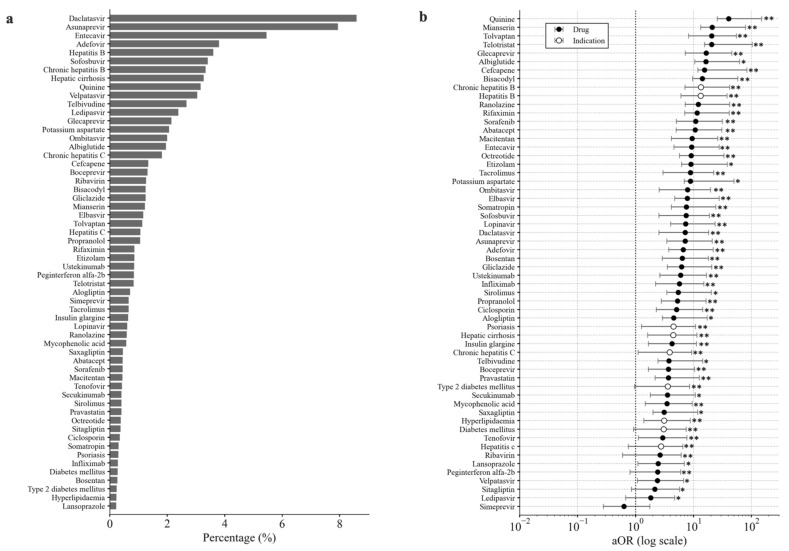
(**a**) Percentage of records with LMT among records with each variate factor. (**b**) Multivariate adjusted odds ratios (aOR) for the reported risk of death shown for each factor. Significant findings (by the Wald test) are indicated with asterisks (* *p* < 0.05, ** *p* < 0.01), and 95% confidence intervals are shown as whiskers.

In the process of constructing the multivariate logistic regression model, approximately half of the drugs were found not to be significantly associated with LMT risk, and were, thus, excluded. The factors applied to the multivariable logistic regression model and the percentage of patients with LMTs for each variate factor are shown in Figure 4a. The multivariate aORs and 95% confidence intervals of 49 drugs and 11 indications are shown in Figure 4b. After the multivariate adjustment, “quinine” (adjusted odds ratio 40.55, 95% CI (14.93–110.11)), “mianserin” (adjusted odds ratio 21.11, 95% CI (7.77–57.33)), “tolvaptan” (adjusted odds ratio 20.66, 95% CI (12.47–34.24)), “telotristat” (adjusted odds ratio 20.62, 95% CI (5.11–83.13)), and “glecaprevir” (adjusted odds ratio 16.50, 95% CI (9.30–29.28)) were the greatest predictors of LMT (Figure 4). “simeprevir” was adopted for the final multivariate logistic model, but was not a significant factor.

### 3.4. Profiling of MIEs Associated with LMT Induction

The principal objectives of our research were to characterize LMT-inducing drug-specific MIEs detected from the adverse event database, and to provide toxicological insights.

Excluding platinum drugs and high-molecular-weight drugs from the 111 drugs that were collected, as described in Section 2.9, Toxicity Predictor could estimate the predicted MIE activity values for 107 drugs.

A total of 54 MIEs were assessable. The six MIEs that showed significant differences were “estrogen receptor-β antagonist” activity, “estrogen-related receptor antagonist” activity, “ATAD5 genotoxic inducer” activity, “estrogen-related receptor agonist” activity, “vitamin D receptor agonist” activity, and “thyrotropin-releasing hormone receptor” agonist activity (Table 6). A complete list of RORs for LMT-inducing drugs and MIE activities is available in Table S3.

Of note, the four representative drugs detected in the multivariate analysis, i.e., quinine, mianserin, telotristat, and tolvaptan, which showed a potential signal for LMTs, exhibited some of these biological features. Quinine exhibited agonist activity against thyrotropin-releasing hormone receptor (TRHR). Mianserin showed agonist activity against ATAD5, estrogen-related receptor (ERR), and TRHR, and showed antagonist activity against ERR. Tolvaptan showed agonist activity against ATAD5, ERR, and TRHR. Telotristat showed agonist activity against ATAD5 and ERR.

### 3.5. Comparison with the Latest DILIst Dataset

Our MIE dataset showed an overall 68% overlap with the DILIst dataset. The overlap rate of the drugs belonging to the LMT-inducing class in the MIE dataset, with the positive drugs in the DILIst dataset, was 36%. In addition to the partial concordance between the drugs in the latest DILIst dataset and LMT-inducing drugs, these results revealed a potential new class of LMT-inducing drugs that are distinct from DILI-inducing drugs.

**Figure 5:**
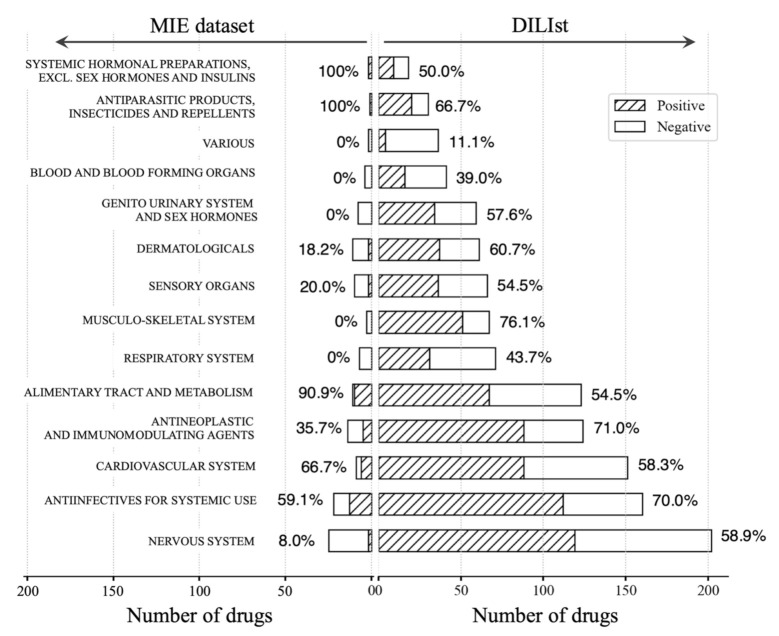
ATC distribution of LMT-inducing drugs and comparison with the DILIst dataset. Comparison of DILI-negative (white bars) and DILI-positive drugs (hatching bars) between the MIE and DILIst dataset at the anatomical group level. Percentages depict the ratio of positive drugs in each anatomical class.

The analysis of the anatomical group, which is the top level of the ATC Classification System (Figure 5), revealed that both the DILIst and MIE datasets covered all 14 anatomical categories. The ATC anatomical categories that were abundant in DILIst partially did not exist in the LMT-positive drugs of the MIE dataset. Particularly in the MIE dataset, the categories of various agents for the blood and blood-forming organs, genitourinary system, sex hormones, musculoskeletal system, and respiratory system did not include LMT-inducing drugs. LMT-inducing drugs were mainly localized to the categories of systemic hormonal preparations (excluding sex hormones and insulins), antiparasitic products, insecticides, repellents, alimentary tract and metabolism, cardiovascular system, and anti-infectious agents for systemic use.

The analysis of the therapeutic group, which is the first subgroup in the ATC Classification System, revealed a new category of LMT-inducing drugs, which were other alimentary tract and metabolism products that were distinct from the drugs in the DILIst dataset, and telotristat (aOR: 20.62) in the MIE dataset belonged to this category.

## 4. Discussion

Recent studies have hypothesized that certain pharmacological characteristics are associated with DILI, based on evidence from FAERS [45]. In the present study, which identified LMT-inducing drugs from the FAERS database, we found that there was concordance between LMT-inducing drugs and known DILI-positive drugs. Based on our analyses, we hypothesize that NR/SR modulation underlies the pharmacological basis of drug-induced LMT.

LMT-inducing drugs had partial concordance with known DILI-positive drugs in the DILIst dataset. Moreover, some of the LMT-inducing drugs, such as telotristat, belonged to a new therapeutic category that was distinct from that in the latest DILIst dataset. A key finding was the identification of quinine, mianserin, telotristat, and tolvaptan as new drugs, with a potential signal for LMTs by multivariate analysis. Of these, only tolvaptan belonged to the DILIst dataset. However, a potential signal for DILI has been demonstrated for quinine, mianserin, and telotristat in several studies, supporting the hypothesis that DILI is linked to LMTs [46,47,48,49,50].

The four representative drugs with a potential signal for LMTs exhibited several biological features. Quinine showed agonist activity against TRHR. Mianserin exhibited agonist activity against ATAD5, ERR, and TRHR, and showed antagonist activity against ERR. Tolvaptan showed agonist activity against ATAD5, ERR, and TRHR, whereas telotristat showed agonist activity against ATAD5 and ERR.

### 4.1. FAERS Database

In this study, our analysis included records from a patient population that was adjusted for consistency and pseudo-signals in the time series of duplicate reports and ADE reports. Therefore, the drugs suspected of inducing LMTs identified in our study are more reliable signals than could be detected from the raw FAERS data.

### 4.2. Suspected Drugs and Indications

The major classes of drugs suspected of inducing LMTs were prescribed for hepatitis B and hepatitis C. HCV infection is a significant predictor of HCC development. Direct-acting antiviral (DAA) agents against HCV achieve sustained viral response rates of more than 90% in treated patients, and have an excellent safety profile [34]. However, some investigators have reported that DAAs were related to an unexpectedly high incidence of de novo HCC [35,36,51], reflecting a controversial relationship between DAA use and HCC incidence. Villani et al. [52] proposed several molecular mechanisms involved in HCC recurrence after treatment with DAAs, including immune cell dysfunction, altered cytokine networks, and activation of angiogenesis; however, the risk of HCC recurrence with DAAs, and the mechanisms involved, have not been definitively determined. In the present study, multivariate analysis showed that DAA agents such as glecaprevir had higher aORs than peg-interferon alpha-2b. However, a retrospective multicenter cohort trial by Bielen et al. found that there was no difference in the rate of early occurrence of new HCC among patients treated with DAA agents without pegylated interferon (PEG-IFN), and the rate of early HCC recurrence in patients treated with DAA agents without PEG-IFN was higher than that in patients treated with PEG-IFN+DAA. However, the patients treated with DAA agents without PEG-IFN were at a higher risk of HCC at baseline [53]. Therefore, the LMT signal of DAA agents identified in the present study remains hypothetical.

Hepatitis B, hepatitis C, liver transplantation, use of immunosuppressive drugs, and other liver diseases were identified as indications suspected to be associated with LMT induction. Among the applicable diseases for which signals were detected, we found a strong association with hepatocarcinoma in chronic hepatitis B, chronic hepatitis C, hepatitis C, growth hormone deficiency, psoriasis, diabetes mellitus, type 2 diabetes, and hyperlipidemia, through a detailed literature review. Chronic HBV infection accounts for at least 50% of all HCC cases worldwide. In chronic HBV infections, recurrent hepatitis caused by the host immune response may lead to liver fibrosis and cirrhosis, acceleration of the hepatocyte metabolic turnover rate, and accumulation of mutations [54]. Growth hormone deficiency, psoriasis, insulin resistance, and hyperlipidemia are risk factors associated with NAFLD [44]. In recent years, a growing body of epidemiological evidence suggests that NAFLD is a major etiological factor underlying many cases of HCC [55]. The findings of our study support this relationship, as these patient background indications were strongly associated with HCC.

### 4.3. Multivariate Logistic Regression

The major class of drugs suspected to induce LMTs was that used for the treatment of infectious liver diseases. Additionally, in the signal detection for indications related to LMT induction, the patient backgrounds related to LMT were infectious liver disease, cirrhosis, diabetes, metabolic syndrome, and endocrine disorders, which represent high risks for HCC development. Therefore, these patient backgrounds were considered to be at higher risk for LMT ADEs, resulting in signal detection of the drugs used to treat these diseases.

Thus, we conducted a multiple logistic regression analysis, conditioned on potential confounders, to calculate adjusted odds ratios for the influence of these factors on the outcome [56,57]. The drug signal results obtained using the adjusted odds ratios were expected to provide a more accurate signal for the ROR assessment, and were used for further evaluations.

### 4.4. Potential MIEs Specific to Suspected LMT-Inducing Drugs

The following six MIEs were statistically identified as MIEs specific to suspected LMT-inducing drugs: estrogen receptor-β (ER-β) antagonist, ERR agonist and antagonist, ATAD5 genotoxic inducer, vitamin D receptor (VDR) agonist, and TRHR agonist.

ER-β is a nuclear hormone receptor that plays an important role in development, metabolic homeostasis, and reproduction. In human-derived hepatoma cells, ER-β directly downregulates peroxisome proliferator-activated receptor-α (PPARα) gene expression and inhibits nuclear translocation, suppressing proliferation and inducing apoptosis [58,59]. Therefore, it is reasonable to expect that ER-β antagonists could be involved in hepatocarcinogenesis via growth promotion and apoptosis inhibition. Furthermore, estrogen receptor function has been shown to contribute to the inhibition of NASH and NAFLD [60]. Animal models have shown that the direct modulation of liver bile acid receptor function, by ER-β agonists, or indirect modulation, by adipose suppression, could provide therapeutic benefits for NASH [61]. Therefore, ER-β antagonists may act on bile acid and lipid regulation, negatively affecting NASH and NAFLD, which are the major underlying etiologies of HCC.

ERR plays an important role in cellular energy metabolism regulation. ERR-α shares sequence similarity with ER-α, but is not activated by endogenous estrogen. ERR-α was previously identified as an adverse marker for breast cancer progression, indicating poor prognoses for ERR-α-positive tumors [62], and increased cancer cell migration, proliferation, and tumor development [63]; thus, this demonstrates that the ERR-α agonist can affect tumorigenesis. Our results showed that ERR antagonist activity is also a potential MIE for drug-induced LMTs. However, associations between ERR antagonists and tumorigenesis/proliferation and LMT induction have not been confirmed, and the relationship between the modulation of energy regulation mechanisms by ERR and the LMT development requires more detailed investigation.

Enhanced levels of genome instability gene 1 (ELG1; human ATAD5) protein levels have been shown to increase in response to DNA damage [64]. Therefore, ATAD5 is a biomarker for identifying genotoxic compounds. ATAD5 expression is significantly higher in human cancers, such as HBV-associated HCC and various HCC cell lines, compared to normal liver cells [65]. Our study showed the relevance of ATAD5 as a potential MIE specific to LMT-inducing drugs. This finding indicates that LMT-inducing drugs contain a significant number of DNA-damage-causing compounds, supporting the possibility that genotoxic compounds are associated with drug-induced LMT development.

In addition to its role in regulating calcium and phosphate metabolism, VDR may also be involved in the development and progression of NASH. The activation of VDR in non-parenchymal cells can protect the liver from overwhelming immune responses, whereas the activation of parenchymal VDR may produce undesirable outcomes via lipid accumulation in the liver [66]. Therefore, VDR agonist activity may induce LMT development by promoting lipid accumulation in the liver.

TRHR is a G-protein-coupled receptor that binds tripeptide thyrotropin-releasing hormone. TRHR is found on the anterior pituitary and has thyrotropin-releasing hormone (TRH) as its ligand. TRH is released from the hypothalamus, acting on the anterior pituitary gland via regulation of thyroid hormones modulated by thyroid-stimulating hormone; their homeostasis is regulated by a feedback mechanism. Mice with a negative mutation of thyroid hormone receptor TRβ, which is mostly expressed in the liver, develop hepatic steatosis, and their livers become significantly larger. Therefore, a relationship between thyroid hormones and hepatic steatosis has been suggested [67]. Our results support the association of LMT induction as a net effect of TRHR agonist activation, a regulator of thyroid hormones.

### 4.5. Limitations

As the FAERS database is a self-reporting system, it contains duplicated reports, biases, and pseudo-signals. Spontaneous reports may be made by drug manufacturers, doctors, patients, and lawyers; therefore, duplicated reports are common and should be removed before analyses. Additionally, the number of ADE reports is the highest for drugs immediately after launch, because they attract more attention, decreasing gradually over time. Furthermore, when safety information is released by the regulatory authorities, the number of reports for the drug in question or similar drugs increases. Therefore, due to this human bias [68], it is difficult to accurately assess adverse event incidence using FAERS. Furthermore, pseudo-signals are likely to occur in FAERS analyses. For example, indications of the drugs used were listed as ADEs due to insufficient efficacy of the drugs used for that therapeutic purpose, which could cause ADE pseudo-signals. Alternatively, ADE pseudo-signals may be detected when apparent ADEs are observed with commonly used concomitant drugs [25].

Further, MIE activities of the classes of drugs suspected to induce LMTs and those not suspected to induce LMTs were calculated using the QSAR model. Although QSARs enable faster in silico screening for groups of compounds that are difficult to assay directly, large numbers of compounds, and large numbers of toxicity targets, the possibility of false positives and false negatives, due to the QSAR models, cannot be ruled out.

Considering these characteristics of the FAERS database and QSAR predictions, this study analyzed a cleaned-up patient population, in terms of duplicate reports, consistency of ADE reporting time series, and pseudo-signals, and the MIEs specific to the suspected LMT-inducing drug classes that were ultimately identified were also supported by the literature.

## 5. Conclusions

In this study, we applied adverse event databases and toxicity prediction tools to detect potential MIEs that are specific to LMT-inducing drugs. The purpose of this study was to provide findings that can be used to prioritize MIEs in the context of drug development, research, and regulatory decision making. Especially, four representative LMT-inducing drugs, i.e., quinine, mianserin, telotristat, and tolvaptan, shared potential MIEs, including agonist or antagonist activity against ATAD5, ERR, and TRHR. These MIEs can negatively affect drug-induced LMTs. Moreover, the potential MIEs associated with LMTs identified in the present study were supported by studies demonstrating endocrine disruption and cellular stress mediated by these drugs. Therefore, the results of this study may be useful for decision-making applications and the understanding of predictors.

## Figures and Tables

**Table 1:** Preferred terms for liver malignant tumors.

Preferred Terms (PTs)	Source
Cholangiosarcoma	Standardized MedDRA Queries version 22.0
Hepatic angiosarcoma	
Hepatic cancer	
Hepatic cancer metastatic	
Hepatic cancer recurrent	
Hepatic cancer stage I	
Hepatic cancer stage II	
Hepatic cancer stage III	
Hepatic cancer stage IV	
Hepatobiliary cancer	
Hepatobiliary cancer *in situ*	
Hepatoblastoma	
Hepatoblastoma recurrent	
Hepatocellular carcinoma	
Liver carcinoma ruptured	
Mixed hepatocellular cholangiocarcinoma	
Hepatic neoplasm malignant	Manual
Hepatic neoplasm malignant non-resectable	
Hepatic neoplasm malignant recurrent	
Hepatic neoplasm malignant resectable	
Carcinoma hepatocellular	

**Table 2:** Contingency table for calculating reporting odds ratios. *a*, number of LMT reports in exposed group; *b*, number of no-LMT reports in exposed group; *c*, number of LMT reports in non-exposed group; *d*, number of no-LMT reports in non-exposed group. The subscript “drug” means that the exposure factor is drug use. The subscript “ind” means that the exposure factor is indication (diagnoses).

		Exposure Factors			
		Drug		Indication	
		Exposed	Non-Exposed	Exposed	Non-Exposed
**Liver** **Malignant Tumors (LMT)**	with LMT	$a_{drug}$	$c_{drug}$	$a_{ind}$	$c_{ind}$
	with no-LMT	$b_{drug}$	$d_{drug}$	$b_{ind}$	$d_{ind}$

**Table 3:** Contingency table for calculating reporting odds ratios. *a*, number of LMT-inducing drugs in MIE active drugs; *b*, number of non-LMT-inducing drugs in MIE active drugs; *c*, number of LMT-inducing drugs in MIE inactive drugs; *d*, number of non-LMT-inducing drugs in MIE inactive drugs.

		MIEs	
		MIE Active	MIE Inactive
**Drug Class**	LMT-inducing	$a_{MIE}$	$c_{MIE}$
	non-LMT-inducing	$b_{MIE}$	$d_{MIE}$

**Table 4:** Top 10 drugs associated with the development of liver malignant tumors.

Drug	ROR	95% CI	*p*-Value
Daclatasvir	77.73	62.60–96.52	<0.0001 **
Asunaprevir	70.77	45.95–109	<0.0001 **
Entecavir	48.25	31.19–74.66	<0.0001 **
Cinnamomum cassia	35.89	13.96–92.27	<0.0001 **
Adefovir	33.87	18.25–62.84	<0.0001 **
Sofosbuvir	31.25	26.74–36.53	<0.0001 **
Quinine	29.58	12.63–69.3	<0.0001 **
Panax ginseng	28.15	11.00–72.08	<0.0001 **
Atractylodes lancea	27.38	9.44–79.46	0.0003 *
Velpatasvir	26.52	16.86–41.69	<0.0001 **

* *p* < 0.05, ** *p* < 0.0001 (Fisher’s exact test) 95% CI, 95% confidence interval of ROR.

**Table 5:** Top 10 indications associated with liver malignant tumors.

Indication	ROR	95% CI	*p*-Value
Hepatitis B	40.94	28.89–58.02	<0.0001 **
Chronic hepatitis B	38.53	21.87–67.88	<0.0001 **
Hepatic cirrhosis	36.97	25.83–52.93	<0.0001 **
Liver transplant	22.94	14.43–36.45	<0.0001 **
Chronic hepatitis C	22.01	18.67–25.95	<0.0001 **
Prophylaxis against transplant rejection	13.79	8.80–21.61	<0.0001 **
Immunosuppressant drug therapy	13.52	5.82–31.39	<0.0001 **
Hepatitis c	13.04	11.14–15.28	<0.0001 **
Fluid retention	12.82	5.52–29.76	<0.0001 **
Liver disorder	12.48	3.58–43.45	0.0179 *

* *p* < 0.05, ** *p* < 0.0001 (Fisher’s exact test) 95% CI, 95% confidence interval of ROR.

**Table 6:** Molecular initiating events (MIEs) related to liver malignant tumor-inducing drugs.

Molecular Initiating Events	Activity Type	ROR	95% CI	*p*-Value
Estrogen Receptor-β	antagonist	8.08	0.91–71.73	0.040 *
Estrogen-Related Receptor	antagonist	4.65	1.72–12.61	0.002 *
p53	agonist	3.85	0.94–15.80	0.087
Aromatase	antagonist	3.21	0.76–13.60	0.155
ATAD5	genotoxic inducer	2.98	1.30–6.82	0.012 *
Estrogen-Related Receptor	agonist	2.96	1.33–6.56	0.010 *
Vitamin D Receptor	agonist	2.72	1.15–6.39	0.030 *
Thyrotropin-Releasing Hormone Receptor	agonist	2.69	1.22–5.95	0.018 *
Peroxisome Proliferator-Activated Receptor-γ	antagonist	2.52	1.00–6.36	0.060
Human Pregnane X Receptor	agonist	2.40	0.83–6.88	0.113

* *p* < 0.05 (Fisher’s exact test) 95% CI, 95% confidence interval of ROR.

## Supplementary Materials

The following are available online at https://www.mdpi.com/article/10.3390/biom11070944/s1: Table S1: signal-detected LMT-related indications; Table S2: signal-detected LMT-related drugs; Table S3: molecular initiating events (MIEs) related to liver malignant tumor-inducing drugs; Supplemental Material 1: example of preprocesses FAERS database; Supplemental Material 2: list of SMILES structures and MIE activities; Supplemental Information: the details of material and methods.
