# Supplementary material for: Molecular Initiating Events Associated with Drug-Induced Liver Malignant Tumors: An Integrated Study of the FDA Adverse Event Reporting System and Toxicity Predictions
Source: Biomolecules. 2021 Jun 25;11(7):944. doi: 10.3390/biom11070944 (PMC8301945; doi:10.3390/biom11070944)
Supplement: Supplementary file 1 [file biomolecules-11-00944-s001.zip › Supplemental Information.pdf]

Supplemental Information

Patient Information Extraction

(I) Identification of duplicates and exclusion of duplicates.

We extracted the following five subsets from the FAERS database: the demographic table, therapy table, indication table, drug table, and reaction table. Duplicate records were eliminated for each of the five subsets, respectively (Figure SI 1). In the demographic table, reports with the same primary ID and event date were considered duplicate records, which were integrated into a single record. Similarly, in the therapy table, duplicates were identified according to primary ID, drug sequence, and medication start and end dates. In the indication table, duplicates were identified according to primary ID, drug sequence, and indication PT. In the drug table, duplicates were identified according to primary ID, drug sequence, and generic drug name. Finally, in the reaction table, duplicates were eliminated according to primary ID and adverse event PT.

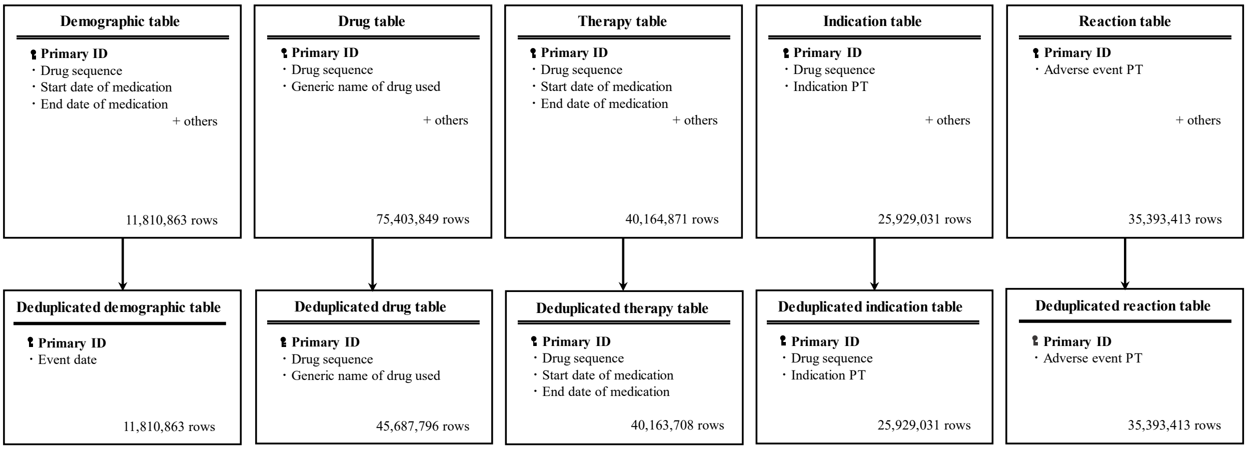

Figure S1. Deduplication of subsets of FAERS database.

(II) Filtering patient information and exclusion.

The deduplicated tables were then merged and integrated into a single table. Next, to extract reports from the integrated FAERS data table that were consistent with the reported ADEs and the drug treatment time series, we extracted records that satisfied the condition that the ADE report date was within the period between the medication start and end dates using the following equation (Eq. 1). Based on Eq. S1, the unique records were narrowed down from 10,181,170 to 1,410,606 as per primary ID.

$$DT_{start} \leq DT_{event} \leq DT_{end} \quad \text{(S1)}$$

Where, DT_{start} is the date the medication was started, DT_{end} is the date the medication was stopped, and DT_{event} is the date of the ADE reported. Records that were missing DT_{start}, DT_{end}, or DT_{event} were excluded. Cases in which indications of the drugs used were reported as ADEs due to insufficient efficacy of the drugs used for that therapeutic purpose could cause a pseudo-signal [25] for drug-induced LMT. Therefore, when records had PTs of indication that belonged to the set of LMT-related PTs, the records were excluded from the data table. Finally, binary information was added to the table to indicate whether the ADE PT was an LMT-related PT. After these adjustments, the data table, "All data table" (Figure S2), contained 8,158,409 rows of information on the primary ID, ADEs, drugs used, indications, medication start and end dates, and ADE report dates without omission. The example of "All data table" can be available from supplemental material 2. To examine drugs suspected of causing LMTs or indications (diagnoses), two data tables, "Table for the analysis of suspected medication" and "Table for the analysis of suspected indication," were created from the "All data table." In the "Table for the analysis of suspected medicines", duplicates were identified according to primary ID, generic name of the drug used, and binary code for LMT. In "The table for the analysis of suspected indications", duplicates were identified according to primary ID, indication PT, and binary code for LMT.

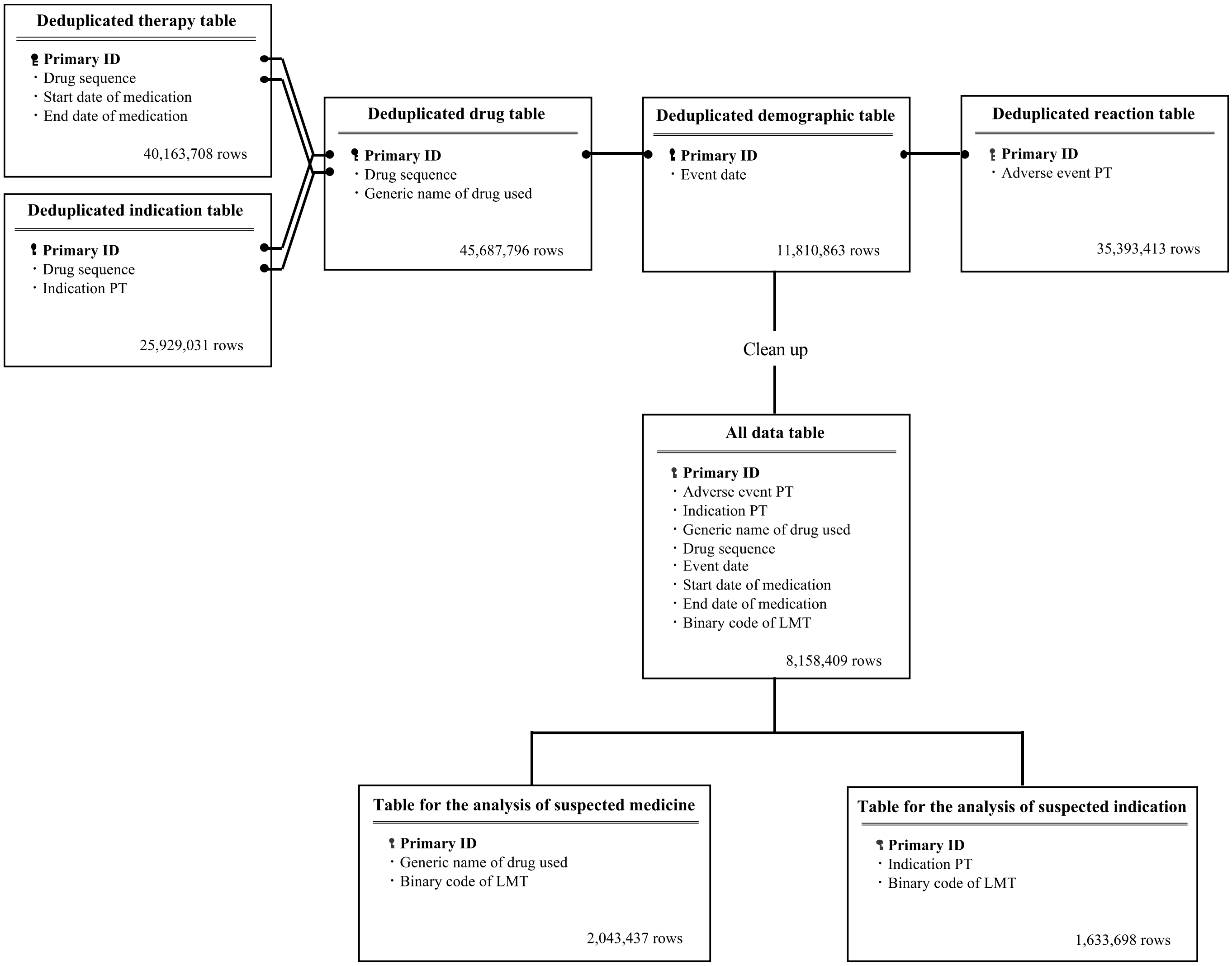

Figure S2. Data table creation schematic, including the names of the data tables used for processing and the names of the processed data tables, the information in each data table, the number of rows in the data table, and the processing flow of the data tables.

Selection of Indications for Multivariate Logistic Regression Model Covariates

The variable selection was conducted to determine whether the risk of patient background to LMT was definitively supported by evidence; therefore, backgrounds that were “not definitively supported by evidence” were discarded. This is because drugs that were used under patient backgrounds which were not definitively supported of the association of LMTs by evidence can themselves essentially belong to the pharmacological class of LMT-inducing drugs. Therefore, "not definitively supported by evidence" was unclear as they could act as confounders of LMTs. Considering this concern and to adjust for potential confounders of LMT-inducing drugs, it was deemed reasonable to conduct multivariate analysis by employing background diseases with known associations with LMT as potential confounders.

Eleven indications were selected and applied in the multivariate logistic regression model: hepatitis B; chronic hepatitis B; hepatic cirrhosis; chronic hepatitis C; hepatitis C; liver disorder; growth hormone deficiency; psoriasis; diabetes mellitus; type 2 diabetes mellitus; and hyperlipidaemia. Hepatitis C virus (HCV)- and hepatitis B virus (HBV)-related infectious intercurrent diseases, e.g., hepatitis B, chronic hepatitis B, chronic hepatitis C, and hepatitis C are major factors in HCC; hepatic cirrhosis is the premalignant state of HCC; growth hormone deficiency, psoriasis, insulin resistance, such as diabetes mellitus and type 2 diabetes mellitus, and hyperlipidemia are risk factors associated with NAFLD as a risk for HCC [34-36]. Immunosuppressant drug therapy, immunosuppression, liver transplant, pain management, and prophylaxis against transplant rejection were not adopted because these were therapeutic methods, not pathologies. Moreover, angina pectoris, ascites, carcinoid syndrome, cardiovascular disorder, cancer pain, constipation, breakthrough pain, dyspepsia, fluid retention, gastric ulcer, HIV infection, hypokalemia, prophylaxis against gastrointestinal ulcer, pulmonary arterial hypertension, thrombocytopenia, and rheumatoid arthritis were not

adopted because their associations with LMT were not definitively supported by evidence. Discarded indications were summarized in Table S4.

Table S4. Discarded indications.

**Indications**
Liver transplant
Prophylaxis against transplant rejection
Immunosuppressant drug therapy
Fluid retention
Ascites
Breakthrough pain
Carcinoid syndrome
Thrombocytopenia
Cardiovascular disorder
Cancer pain
Immunosuppression
Hypokalaemia
Gastric ulcer
Pain management
Angina pectoris
Prophylaxis against gastrointestinal ulcer
Dyspepsia
Pulmonary arterial hypertension
HIV infection
Constipation
Rheumatoid arthritis
