# Supplementary material for: Molecular Initiating Events Associated with Drug-Induced Liver Malignant Tumors: An Integrated Study of the FDA Adverse Event Reporting System and Toxicity Predictions
Source: Biomolecules. 2021 Jun 25;11(7):944. doi: 10.3390/biom11070944 (PMC8301945; doi:10.3390/biom11070944)
Supplement: Supplementary file 1 [file biomolecules-11-00944-s001.zip › Supplemental Results.pdf]

Table S1. Signal-detected LMT-related indications.

Indication	Odds Ratio	95% Confidence Interval	*p*-Value
Hepatitis B	40.94	28.89–58.02	<.0001**
Chronic hepatitis B	38.53	21.87–67.88	<.0001**
Hepatic cirrhosis	36.97	25.83–52.93	<.0001**
Liver transplant	22.94	14.43–36.45	<.0001**
Chronic hepatitis C	22.01	18.67–25.95	<.0001**
Prophylaxis against transplant rejection	13.79	8.80–21.61	<.0001**
Immunosuppressant drug therapy	13.52	5.82–31.39	<.0001**
Hepatitis C	13.04	11.14–15.28	<.0001**
Fluid retention	12.82	5.52–29.76	<.0001**
Liver disorder	12.48	3.58–43.45	0.0179*
Ascites	11.54	4.02–33.11	0.0038*
Breakthrough pain	11.47	3.30–39.93	0.0209*
Carcinoid syndrome	11.05	3.18–38.46	0.0224*
Thrombocytopenia	8.89	3.10–25.49	0.0077*
Cardiovascular disorder	8.68	3.43–21.97	0.0020*
Cancer pain	8.42	4.82–14.73	<.0001**
Immunosuppression	7.17	3.09–16.6	0.0012*
Hypokalaemia	6.29	2.20–18.00	0.0193*
Gastric ulcer	5.75	2.48–13.30	0.0031*
Pain management	5.50	1.92–15.73	0.0272*
Angina pectoris	4.50	2.08–9.73	0.0038*
Growth hormone deficiency	4.35	1.52–12.44	0.0485*
Psoriasis	3.35	2.58–4.35	<.0001**
Prophylaxis against gastrointestinal ulcer	3.31	1.53–7.15	0.0156*
Diabetes mellitus	3.09	2.30–4.15	<.0001**
Type 2 diabetes mellitus	2.73	2.12–3.51	<.0001**
Hyperlipidaemia	2.67	1.56–4.57	0.0023*
Dyspepsia	2.51	1.22–5.14	0.0336*
Pulmonary arterial hypertension	2.42	1.62–3.61	0.0002*
HIV infection	2.01	1.25–3.21	0.0102*
Constipation	2.00	1.09–3.66	0.0480*
Rheumatoid arthritis	1.33	1.03–1.73	0.0421*

*: $p < 0.05$, **: $p < 0.0001$.

Table S2. Signal-detected LMT-related drugs.

Drug	Odds Ratio	95% Confidence Interval	*p*-Value
Daclatasvir	77.73	62.60–96.52	<.0001**
Asunaprevir	70.77	45.95–109	<.0001**
Entecavir	48.25	31.19–74.66	<.0001**
Cinnamomum cassia	35.89	13.96–92.27	<.0001**
Adefovir	33.87	18.25–62.84	<.0001**
Sofosbuvir	31.25	26.74–36.53	<.0001**
Quinine	29.58	12.63–69.3	<.0001**
Panax ginseng	28.15	11.00–72.08	<.0001**
Atractylodes lancea	27.38	9.44–79.46	0.0003*
Velpatasvir	26.52	16.86–41.69	<.0001**
Poria cocos	25.50	9.97–65.19	<.0001**
Telbivudine	24.82	10.62–58.01	<.0001**
Potassium aspartate	21.56	6.14–75.70	0.0065*
Docosahexaenoic acid	20.91	5.96–73.40	0.0069*
Ledipasvir	20.90	15.8–27.63	<.0001**
Glycyrrhiza spp.	19.88	7.80–50.66	<.0001**
Glecaprevir	18.81	11.53–30.69	<.0001**
Pibrentasvir	18.81	11.53–30.69	<.0001**
Albiglutide	18.42	7.23–46.92	0.0001*
Paritaprevir	17.42	13.15–23.08	<.0001**
Ombitasvir	17.36	13.10–23.01	<.0001**
Dasabuvir	15.70	11.43–21.57	<.0001**
Cefcapene	14.06	4.03–49.10	0.0144*
Levomefolic acid	13.10	3.75–45.70	0.0164*
Tocopherol	12.08	4.76–30.65	0.0006*
Bisacodyl	11.81	4.66–29.98	0.0007*
Enterococcus faecalis	11.64	3.34–40.59	0.0204*
Mianserin	11.60	4.57–29.42	0.0007*
Boceprevir	11.56	6.75–19.82	<.0001**
Ribavirin	11.53	9.96–13.34	<.0001**
Gliclazide	11.31	5.50–23.27	<.0001**
Cysteine	11.27	3.23–39.28	0.0217*
Elbasvir	10.80	4.65–25.07	0.0002*
Grazoprevir	10.78	4.64–25.02	0.0002*
Ursodeoxycholic acid	9.95	5.56–17.83	<.0001**
Clostridium butyricum	9.93	3.46–28.50	0.0058*
Eicosapentaenoic acid	9.89	3.45–28.40	0.0058*
Tolvaptan	9.87	6.15–15.83	<.0001**
Propranolol	9.40	5.25–16.84	<.0001**
Glycyrrhizic acid	9.24	2.65–32.15	0.0311*
Ritonavir	9.19	7.09–11.90	<.0001**
Telotristat	8.78	2.52–30.53	0.0342*
Etizolam	8.40	2.93–24.09	0.009*
Calcitriol	8.19	2.36–28.49	0.0386*
Rifaximin	7.97	3.44–18.48	0.0008*
Valine	7.80	2.24–27.11	0.0422*
Leucine	7.77	2.24–27.01	0.0425*
Thiamine	7.64	4.03–14.50	<.0001**

*: $p < 0.05$, **: $p < 0.0001$.

Table S2. Signal-detected LMT-related drugs (continued).

Drug	Odds Ratio	95% Confidence Interval	p-Value
Amino acids nos	7.56	2.64–21.66	0.012*
Isoleucine	7.52	2.16–26.13	0.045*
Ustekinumab	7.45	4.58–12.12	<.0001**
Peginterferon alfa-2b	7.24	5.30–9.89	<.0001**
Alogliptin	6.74	2.66–17.06	0.005*
Sennoside a+b	6.34	2.74–14.69	0.002*
Rabeprazole	6.24	3.29–11.83	<.0001**
Rebamipide	6.06	2.95–12.45	0.0003*
Simeprevir	5.78	3.44–9.70	<.0001**
Tacrolimus	5.67	3.99–8.07	<.0001**
Brotizolam	5.63	1.97–16.13	0.0256*
Insulin glargine	5.60	3.69–8.49	<.0001**
Lactulose	5.57	1.95–15.94	0.0264*
Lopinavir	5.52	2.69–11.33	0.0005*
Ranolazine	5.48	2.37–12.69	0.0038*
Mycophenolic acid	4.98	3.15–7.88	<.0001**
Saxagliptin	4.32	1.71–10.91	0.0219*
Abatacept	4.01	2.19–7.37	0.0004*
Sorafenib	4.01	2.18–7.36	0.0004*
Peginterferon alfa-2a	3.90	2.71–5.61	<.0001**
Loxoprofen	3.89	1.89–7.97	0.0038*
Macitentan	3.86	2.26–6.61	<.0001**
Sirolimus	3.86	1.53–9.75	0.0312*
Glimepiride	3.73	1.72–8.06	0.0091*
Tenofovir	3.71	2.47–5.58	<.0001**
Pravastatin	3.70	1.71–8.01	0.0094*
Octreotide	3.65	1.44–9.22	0.0371*
Secukinumab	3.64	1.92–6.89	0.0016*
Ascorbic acid	3.49	1.61–7.55	0.0123*
Magnesium oxide	3.33	1.62–6.83	0.0085*
Sitagliptin	3.23	2.11–4.94	<.0001**
Spironolactone	3.15	1.81–5.50	0.0009*
Ciclosporin	3.10	1.78–5.41	0.001*
Pyridoxine	3.04	1.32–7.04	0.0372*
Dapagliflozin	2.92	1.35–6.32	0.0263*
Somatropin	2.79	1.29–6.03	0.0319*
Infliximab	2.47	1.54–3.96	0.0011*
Bosentan	2.33	1.31–4.17	0.0197*
Lansoprazole	2.01	1.15–3.51	0.0399*
Metformin	1.86	1.20–2.88	0.0143*

*: $p < 0.05$, **: $p < 0.0001$.

Table S3. Molecular initiating events (MIEs) related to liver malignant tumor-inducing drugs.

AID	Molecular Initiating Events	Activity Type	ROR	95% Confidence Interval	*p*-Value
1259396	Estrogen Receptor-β	antagonist	8.08	0.91−71.73	0.040*
1259403	Estrogen Related Receptor	antagonist	4.65	1.72−12.61	0.002*
720552	p53	agonist	3.85	0.94−15.80	0.087
743139	Aromatase	antagonist	3.21	0.76−13.60	0.155
720516	ATAD5	genotoxic inducer	2.98	1.30−6.82	0.012*
1259404	Estrogen Related Receptor	agonist	2.96	1.33−6.56	0.010*
743241	Vitamin D Receptor	agonist	2.72	1.15−6.39	0.030*
1347030	Thyrotropin Releasing Hormone Receptor	agonist	2.69	1.22−5.95	0.018*
743199	Peroxisome Proliferator-Activated Receptor-γ	antagonist	2.52	1.00−6.36	0.060
1347033	human Pregnane X Receptor	agonist	2.40	0.83−6.88	0.113
743063	Androgen Receptor lbd	antagonist	2.24	0.94−5.32	0.079
743228	Heat Shock Response	activator	2.03	0.43−9.57	0.440
743091	Estrogen Receptor-α full	antagonist	1.91	0.85−4.34	0.145
1259387	Androgen Receptor with antagonist	agonist	1.89	0.82−4.33	0.143
743077	Estrogen Receptor-α lbd	agonist	1.87	0.86−4.07	0.121
1224894	Hypoxia (HIF-1)	agonist	1.87	0.85−4.11	0.160
743054	Androgen Receptor full	antagonist	1.80	0.67−4.670	0.322
1159531	Retinoid X Receptor-α	agonist	1.80	0.69−4.670	0.322
1347034	Caspase-3/7 in HepG2	inducer	1.78	0.82−3.893	0.168
743219	Antioxidant Response Element	agonist	1.77	0.79−3.98	0.213
743053	Androgen Receptor lbd	agonist	1.67	0.75−3.71	0.226
1259402	Estrogen Related Receptor with PGC	agonist	1.63	0.75−3.556	0.238
743122	Aryl Hydrocarbon Receptor	agonist	1.63	0.65−4.05	0.349
743067	Thyroid Receptor	antagonist	1.61	0.71−3.64	0.298
743226	Peroxisome Proliferator-Activated Receptor-δ	antagonist	1.52	0.70−3.32	0.325
743242	Vitamin D Receptor	antagonist	1.47	0.09−24.06	1.000
1259391	Estrogen receptor alpha with antagonist	agonist	1.47	0.09−24.06	1.000
1159528	Activator Protein-1	agonist	1.45	0.67−3.13	0.434
1259248	Estrogen Receptor-α with stimulator	antagonist	1.42	0.64−3.14	0.420
1259401	Estrogen Related Receptor with PGC	antagonist	1.30	0.60−2.83	0.555
720719	Glucocorticoid Receptor	agonist	1.23	0.56−2.72	0.688

*: $p < 0.05$.

Table S3. Molecular initiating events (MIEs) related to liver malignant tumor-inducing drugs (continued).

AID	Molecular Initiating Events	Activity Type	ROR	95% Confidence Interval	*p*-Value
1159518	NF-kB	agonist	1.156	0.40−3.38	0.790
1224893	Constitutive Androstane Receptor	antagonist	1.044	0.310−3.53	1.000
743140	Peroxisome Proliferator-Activated Receptor-γ	agonist	1.027	0.45−2.344	1.000
1347032	Transforming Growth Factor-β	antagonist	0.852	0.36−2.04	0.826
720725	Glucocorticoid Receptor	antagonist	0.707	0.17−2.99	0.735
743227	Peroxisome Proliferator-Activated Receptor-δ	agonist	0.707	0.17−2.99	0.735
1347035	Transforming Growth Factor-β	agonist	0.707	0.17−2.99	0.735
1224892	Constitutive Androstane Receptor	agonist	0.636	0.27−1.50	0.396
743240	Farnesoid-X-Receptor	antagonist	0.473	0.05−4.70	0.644
1224896	histone variant H2AX	agonist	0.473	0.05−4.70	0.644
720637	Mitochondrial Membrane Potential	disruptor	0.447	0.11−1.76	0.353
743239	Farnesoid-X-Receptor	agonist	0.388	0.08−1.96	0.305
1159519	Endoplasmic Reticulum stress response	agonist	0.349	0.04−3.23	0.646
1259247	Androgen Receptor with stimulator	antagonist	0.291	0.06−1.42	0.194
1259392	Sonic Hedgehog signaling (Shh)	antagonist	0.274	0.03−2.43	0.398
743078	Estrogen Receptor-α lbd	antagonist	0.225	0.03−1.94	0.237
1224895	Thyroid Stimulating Hormone Receptor	agonist	0	-	0.269
1259388	Histone Decetylase (HDAC)	antagonist	0	-	0.078
1259395	Thyroid Stimulating Hormone Receptor	antagonist	0	-	0.144
1347031	Progesterone Receptor	antagonist	0	-	1.000
1347036	Progesterone Receptor	agonist	0	-	0.513
1347037	Caspase-3/7 in CHO-K1	inducer	0	-	0.079
1347038	Thyrotropin Releasing Hormone Receptor	antagonist	0	-	0.269
1259390	Sonic Hedgehog signaling (Shh)	agonist	-	-	-
1259394	Estrogen Receptor-β	agonist	-	-	-

*: $p < 0.05$.
